# Resection status and margin control in intraoperative frozen sectioning analysis of oral squamous cell carcinoma

**DOI:** 10.1007/s10006-024-01238-x

**Published:** 2024-03-27

**Authors:** Felix Neumann, Xenia Straub, Friedrich Mrosk, Kerstin Rubarth, Johanna Wolfsberg, Iris Piwonski, Christian Doll, Jan Voss, Max Heiland, Kilian Kreutzer, Steffen Koerdt

**Affiliations:** 1 Department of Oral and Maxillofacial Surgery, Charité Universitätsmedizin Berlin, Corporate Memberember of Freie Universität Berlin, Humboldt-Universität Zu Berlin, and Berlin Institute of Health, Berlin, Germany; 2 Berlin Institute of Health (BIH), Anna-Louisa-Karsch-Straße 2, 10178 Berlin, Germany; 3 Charité - Universitätsmedizin Berlin, Corporate member of Freie Universität Berlin and Humboldt-Universität Zu Berlin, Institute of Biometry and Clinical Epidemiology, Charitéplatz 1, 10117 Berlin, Germany; 4 Charité - Universitätsmedizin Berlin, Corporate member of Freie Universität Berlin and Humboldt-Universität Zu Berlin, Institute of Medical Informatics, Charitéplatz 1, 10117 Berlin, Germany; 5 Department of Pathology, Charité - Universitätsmedizin Berlin, Corporate member of Freie Universität Berlin and Humboldt-Universität Zu Berlin, Charitéplatz 1, 10117 Berlin, Germany

**Keywords:** OSCC, Oral cancer, Intraoperative frozen sectioning, Frozen section biopsy, Margin

## Abstract

**Purpose:**

Intraoperative frozen section analysis (IFSA) is a well-established procedure for determining the intraoperative soft tissue resection status in patients with oral squamous cell carcinoma (OSCC). Margin status is a major predictor of the patient´s outcome, histologically free margins of ≥ 5 mm are demanded. This study evaluates the accuracy of IFSA, the impact of margin status and the impact of intraoperative margin revision on disease-free survival (DFS) and overall survival (OS).

**Methods:**

This retrospective study included 213 patients with OSCC. IFSA results were compared with definitive histopathological reports, Kaplan–Meier analysis was performed. Cut-off values were calculated for resection margins considering known risk factors.

**Results:**

IFSA showed positive margins in 8 cases (3.8%). Kaplan–Meier analysis revealed no significant differences for OS or DFS if R0-status was achieved by initial resection or immediate re-resection.

Final histopathological evaluation revealed false-positive IFSA in 3/8 cases (37.5%) and false-negative IFSA in 1/205 cases (0.5%). Sensitivity was 83.3% and specificity was 98.6%.

Analysis of optimal cut-off values showed no general need for larger resection margins in patients with risk factors. Cut-off values were slightly higher for patients with the risk factor alcohol consumption (7 mm for OS and DFS) or pN + ECS- disease (7 mm for DFS). Optimal cut-off values for tumour-margin-distance were around 6 mm.

**Conclusion:**

IFSA provides a valuable assessment method for intraoperative soft tissue resection margins. Risk factors seemingly do not significantly influence the extent of tumour resection.

## Introduction

Despite interdisciplinary treatment regimens and advancements in multimodal and individualised therapies, the overall prognosis of oral squamous cell carcinoma (OSCC) has not improved over the last decade [1–3]. In early stages, surgical resection with or without adjuvant therapy is still considered to be the gold standard, even when the disease has spread to cervical lymph nodes [4, 5]. However, several prognostic factors for the treatment of OSCC determine the overall prognosis and disease-free survival (DFS). Higher TNM categories correlate with a worse prognosis [6, 7]. In contrast to continuous growth with a clearly definable growth front, discontinuous infiltrative tumour growth leads to a worse prognosis, especially at primary sites such as the tongue and the floor of the mouth [8, 9]. Nevertheless, a sufficient in sano resection (R0) with adequate safety margins is one of the most important prognostic factors in surgically treated OSCC. R0 resection with adequate safety margins has to be the goal of curative surgical treatment. The prognostic relevance of the final resection margin status on overall survival (OS) and DFS has been described independently by several research groups [10, 11].

Intraoperative frozen section analysis (IFSA) is a well-established and widely used practice for examining soft tissue resection margins. It promptly provides information about the intraoperative resection status [12–14]. Nevertheless, there is no consistent approach concerning how to take the samples for frozen sectioning. Both specimen-driven and tumour-defect-driven frozen sectioning are known to be reliable procedures for evaluating intraoperative margins. As previous studies were not able to show a significant difference in OS or DFS, in our clinical practice we routinely perform defect-driven frozen sectioning [15–17].

The threshold for close margin definition has been set at 1–5 mm [12, 13, 18]. However, a close margin or R1 resection has been linked to a significantly worse overall prognosis, whereas the current evidence do not clearly indicate whether a close margin status is an independent risk factor for higher local recurrence rates [19, 20]. Additionally, the impact of other risk factors is still debated. Perineural tumour invasion (PnI) is a modulating factor for the recurrence rate [21]. Nevertheless, achieving R0 resection is commonly accepted to be a main goal in curative-intended surgical treatment of OSCC. IFSA can help to achieve tumour clearance and thus may be able to improve the patient´s prognosis. However, the impact of re-resection after an initial R1 resection status based on IFSA for a patients’ DFS is still debated [12, 22–24].

The aim of this study was to evaluate the accuracy of IFSA. In addition, we compared the prognosis after achieving an R0 status from the initial resection with the prognosis after achieving an R0 status via immediate re-resection. Furthermore, we investigated the prognostic value of the resection status and margin distance provided by IFSA on OS and DFS.

## Material and methods

The current retrospective work analyses a cohort of patients with primary OSCC treated at the Department of Oral and Maxillofacial Surgery of the Charité Universitätsmedizin Berlin between April 2017 and February 2021. The inclusion criteria were (i) curative treatment intention and (ii) intraoperative frozen sectioning during tumour resection. Patients with local recurrences, preoperative irradiation in the head and neck area as well as chemotherapy were excluded from the study. All included patients have received the recommended adjuvant therapy. The R-status in the histopathological report was investigated. Close margin status was defined as resection distance 1 mm < X < 5 mm. R1 status was defined as a (carcinoma-)positive resection margin. Frozen sectioning specimens were collected circularly and from the tumour bed after main tumour resection to evaluate the intraoperative resection status. Sensitivity and specificity were evaluated by referring to the definitive histological evaluation of these specimens. The evaluation was performed for the complete patient cohort and subdivided according to the different intraoral tumour locations. Furthermore, the prognostic value of the resection distance according to IFSA was evaluated by calculating cut-off values. The clinical outcome was investigated in terms of OS and DFS, including locoregional recurrences, lymph node recurrences and the occurrence of metastases and secondary carcinomas defining the end of the DFS interval. Only patients with complete data sets were included.

### IFSA – intraoperative frozen section analysis

The intraoperative consultation of the tissue included the frozen section of the specimen. The specimens were measured and then embedded in a gel-like medium and cooled down using the freezing area of the cryostat microtome or via liquid nitrogen. The specimen was cut into slices of 5 (up to 10) µm, put on glass slides and stained with haematoxylin and eosin (H&E).

After IFSA, all frozen specimens were formalin fixed and paraffin embedded (FFPE) and additional slides of the FFPE tissues were stained with H&E and evaluated.

### Statistical analysis

Data were collected in Microsoft Excel (Microsoft Corporation, Redmond, WA, USA) and analysed with SPSS Statistics (IBM Corporation, Armonk, NY, USA) and R Studio (RStudio Team, Boston, MA, USA). The means and standard deviations (SD) as well as the median and first (q1) and third (q3) quartiles were calculated for metric variables and absolute as well as relative frequencies were determined for categorical data. Categorical variables were compared across the groups by using cross tables and the chi-square tests. Kaplan–Meier analysis was performed for survival analysis, calculating OS and DFS. The log rank test was performed to test relationships between categorical variables and OS or DFS. Sensitivity and specificity were calculated and the optimal cut-off values were obtained by using Youden´s index for receiver operating characteristic (ROC) analyses of logistic regressions regarding OS and DFS. Furthermore, Cox regression was performed adjusted for age, sex and Union for International Cancer Control (UICC) stage. Hazard ratios and their 95% confidence intervals (CI) were calculated. Statistical significance was defined as α = 0.05, p-values and CI were not adjusted for multiplicity due to the exploratory nature of this study. 

## Results

### Patients’ characteristics

This retrospective study included 213 patients (113 [53%] men / 100 [47%] women) with OSCC treated with primary surgical resection at the department of Oral and Maxillofacial surgery at Charité Universitätsmedizin Berlin, Germany. The mean ± SD age was 65 ± 11.6 years (range 26 – 93 years). The median follow-up was 26 months (range 1 – 57 months). The localisation of the main tumour as well as the TNM and UICC status are presented in Table 1.

**Table 1:** Localisation and clinical and pathohistological TNM (8th edition) and UICC-status

Parameter	N	%
Localisation		
Tongue	65	30.5
Floor of mouth	58	27.2
Mandible	53	24.9
Maxilla	21	9.9
Soft palate	1	0.5
Cheek	15	7.0
pT stage		
pT1	65	30.5
pT2	67	31.5
pT3	25	11.7
pT4a	56	26.3
pN stage		
pN0	140	65.7
pN1	24	11.3
pN2a	5	2.3
pN2b	9	4.2
pN2c	3	1.4
pN3b	31	14.6
n.a.*	1	0.5
pM stage		
pM0	209	98.1
pM1	2	0.9
pMx	1	0.5
n.a.*	1	0.5
pUICC		
I	59	27.7
II	43	20.2
III	28	13.1
IVA	52	24.4
IVB	29	13.6
IVC	1	0.5
n.a.*	1	0.5

* In one patient with cN0 status, ND was not performed, due to age and comorbidities

### Intraoperative versus final resection status

The intraoperative resection status was compared with the final resection status of the IFSA specimens. After final histopathological evaluation, there was one false-negative IFSA (0.5%). Surgical re-resection was not performed, neither intraoperatively nor in a second surgery, because microvascular reconstruction had already been in situ for several days. This case was formally treated as an R1-resection and the patient received adjuvant radiation. Due to the patient´s request and patient´s reduced general condition, no systemic chemotherapy was performed. There were also three cases of false-positive IFSA (37.5%). In all of these cases, IFSA could not exclude small branches of tumour in the margins. Therefore, intraoperative re-resection was suggested and performed. After paraffin embedding, clear margins could be identified in the primary IFSA specimens. Overall, the sensitivity for IFSA was 83.3% (95%-CI: [43.7%–97.0%]) and specificity was 98.6% (95%-CI: [97.3%–99.9%]). The characteristics of the patients with false-negative or false-positive IFSA are presented in Table 2.

**Table 2:** Patients’ characteristics with false-negative or false-positive IFSA

Parameter	pTNM	pUICC	IFSA	Final resection status after intraoperative re-resection (if performed)
	T4a N3b M0	IVB	negative	R1
	T4a N0 M0	IVA	positive	R0
	T2 N1 M0	III	positive	R0
	T3 N3b M1*	IVB	positive	R0

* Patient was initially staged as cM0, thoracic metastases were diagnosed after tumour resection was performed in curative intention

### Intraoperative frozen section analysis

#### Resection status

In all cases, frozen sectioning samples were taken separately after the main resection in a circular manner. In a clinical setting a macroscopic margin of 10 mm has to be achieved during tumor resection. The mean amount of frozen sectioning samples was 8 (range 2–28 samples). In total, 1776 defect-driven specimens were evaluated. Overall, there were 8 (3.8%) patients with an R1 resection status according to IFSA. Five (62.5%) of these patients presented with a T4a stage, in one (12.5%) case each with T1, T2 and T3 stage, respectively. Intraoperative re-resection was performed in all of these cases. Therefore, a specimen of approximately 3 mm in size, immediately adjacent to the positive margin, was discarded. Subsequently, a new specimen was collected for the re-evaluation of the margin status. Re-resection was successful in achieving a final R0 resection status in 7 of 8 (87.5%) cases. The resection status based on IFSA and after final histopathological analysis is shown in Table 3.

**Table 3:** Resection status based on IFSA and after final histopathological analysis

Resection status	n IFSA	Final resection status (including intraoperative re-resection)
Positive margin	8 (3.8%)	R1: 1 (12.5%) R0: 7 (87.5%)
Negative margin	205 (96.2%)	R1: 1 (0.5%) R0: 204 (99.5%)

**Fig. 1:**
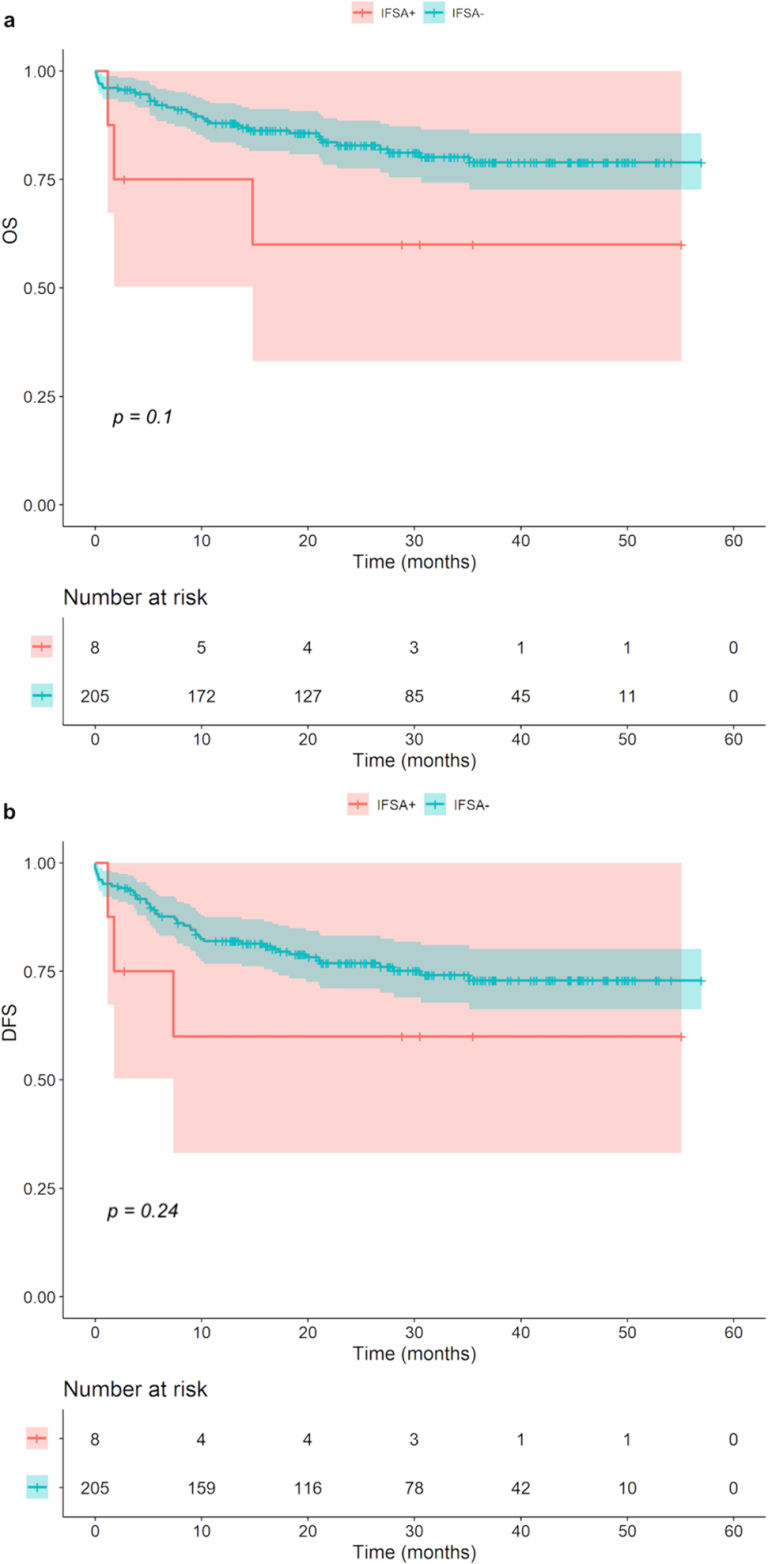
Kaplan–Meier analysis: positive initial IFSA versus negative initial IFSA, regarding OS (**a**) and DFS (**b**)

**Fig. 2:**
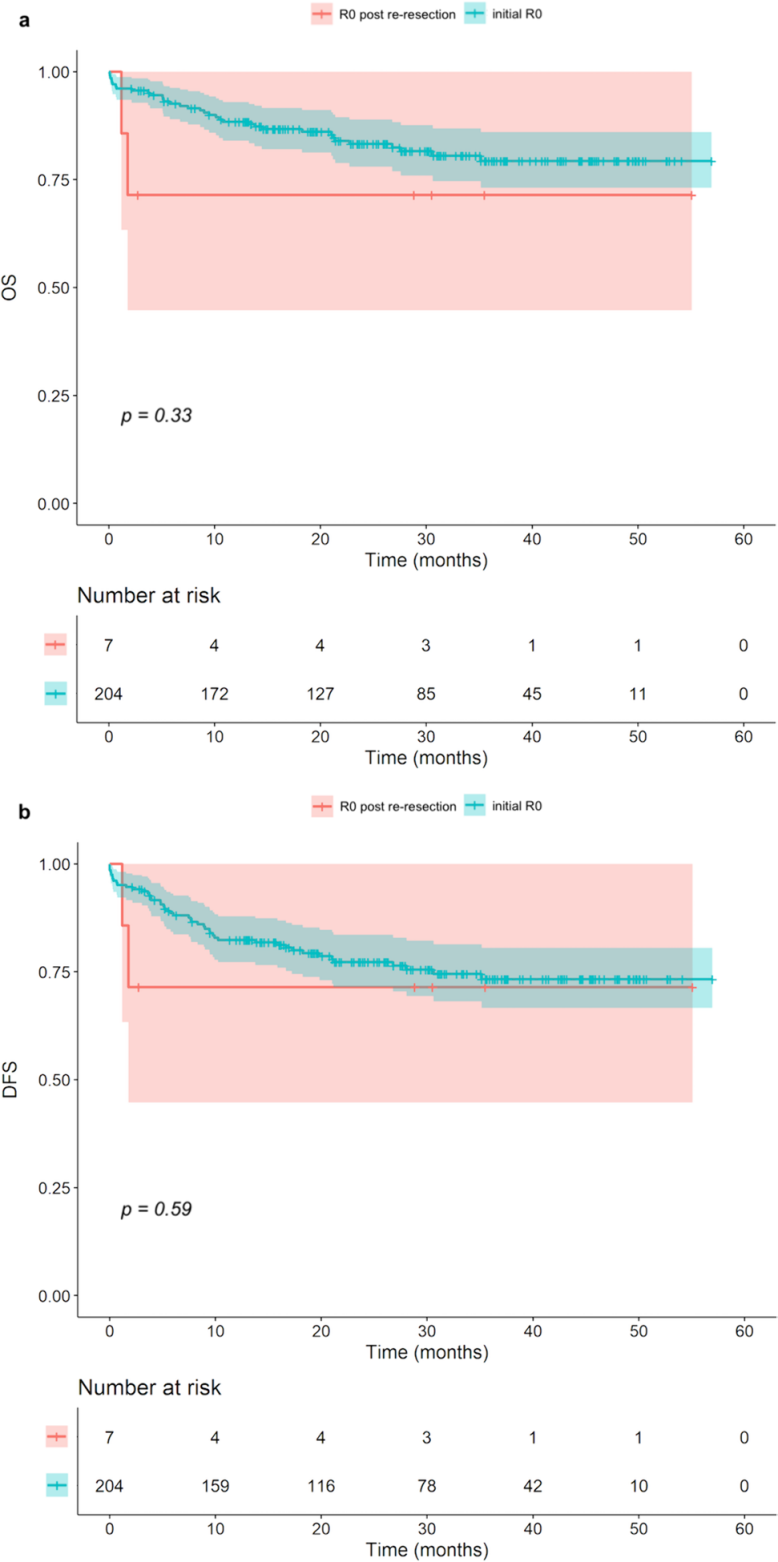
Kaplan–Meier analysis: impact of initial R0 versus R0 after immediate re-resection, regarding OS (**a**) and DFS (**b**)

The Kaplan–Meier analysis was performed to compare the prognostic value of the IFSA. For the OS and the DFS, there was a tendency for poorer survival for patients with positive margins after initial IFSA (before re-resection) compared with those with negative margins after initial IFSA. However, the difference between the groups in OS (*p* = *0.10*) and DFS (*p* = *0.24*), was not statistically significant (Fig. 1). Furthermore, there were no statistically significant differences in OS (*p* = *0.33*) and DFS (*p* = *0.59*) between the group with an initial R0 resection status and the patients that needed immediate re-resection to achieve a final R0 resection status (Fig. 2).

In 52 cases (24.4%), bone invasion of the tumour was confirmed with histopathological evaluation. IFSA (of soft tissue) was positive in 5 patients (9.6%), compared with 3 patients (1.9%) without bone invasion. Successful re-resection could be performed in 4 cases (80.0%). Regarding margin status, there was a statistically significant difference between patients with bone invasion compared with patients without bone invasion (*p* = *0.01*). The resection status of the tumour specimen was also compared (Table 4). Close margin and R1 status were more frequent in the group with bone invasion (*p* = *0.008*). The initial IFSA diagnosis changed in 2 patients (3.8%) in the group with bone invasion and in 2 patients (1.2%) in the group without bone invasion. There was no statistically significant difference (*p* = *0.23*) between the groups regarding the change in the IFSA diagnosis. The final resection status for patients with and without bone invasion is also presented in Table 4. There was a statistically significant difference in the final resection status between the groups (*p* = *0.03*), indicating a tendency for closer overall margins in patients with bone invasion. There were two patients with an R1 margin status after the final histological examination in the bone invasion group. In one of these cases, the positive resection margin was a bone margin, in the other case, the positive margin was found in soft tissue.

**Table 4:** Resection status after initial tumour resection

	Final resection status (including frozen section specimens)				Resection status main tumour specimen		
	> 5 mm	5 mm	< 5 mm	R1	R0	Close margin	R1
Overall	158 (74.2%)	35 (16.4%)	18 (8.5%)	2 (0.9%)	65 (30.5%)	124 (58.2%)	24 (11.3%)
Without bone infiltration	124 (77.0%)	26 (16.2%)	11 (76.8%)	0 (0%)	58 (36.0%)	87 (54.0%)	16 (10.0%)
Bone infiltration	34 (65.4%)	9 (17.3%)	7 (13.5%)	2 (3.8%)	7 (13.5%)	37 (71.1%)	8 (15.4%)

**Fig. 3:**
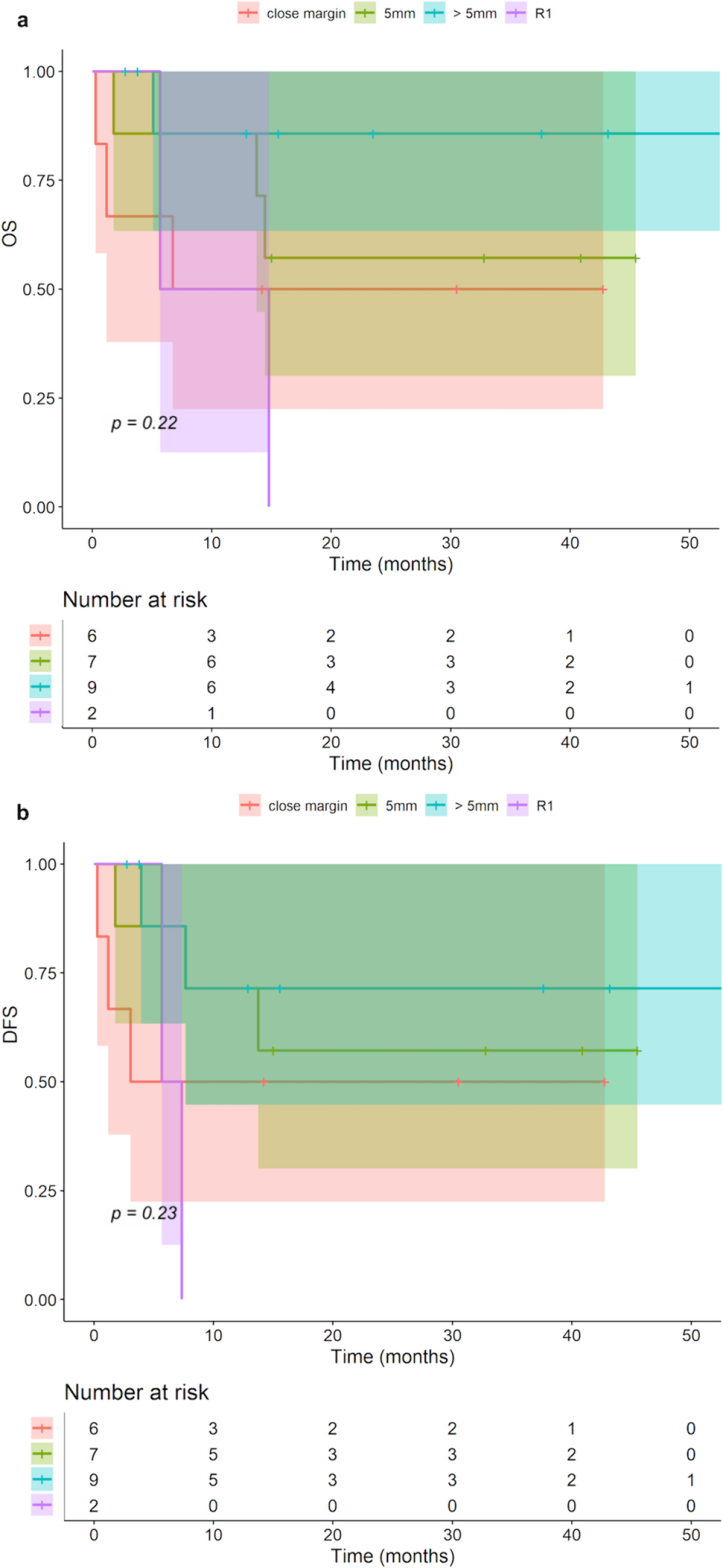
Kaplan–Meier analysis: final resection status regarding OS (**a**) and DFS (**b**)

OS and DFS were compared regarding the final resection status: there were no statistically significant differences. Nevertheless, there was a tendency that resection margins ≤ 5 mm could be associated with a poorer OS and DFS (Fig. 3).

#### Resection distance

Mean resection distance was 7.3 ± 3.3 mm, the median was 6 mm (q1 = 5, q3 = 9). The optimal cut-off values were 6 mm for OS (area under the curve [AUC] = 0.62; sensitivity = 0.78; specificity = 0.44) and 6 mm for DFS (AUC = 0.61; sensitivity = 0.78; specificity = 0.41).

Furthermore, cut-off values were determined considering known risk factors (Table 5).

**Table 5:** Cut-off values for risk factors

Risk factor	Cut-off value (mm)	AUC	Sensitivity	Specificity
Smoking				
OS (smoker)	7	0.59	0.51	0.65
OS (non-smoker)	6	0.64	0.80	0.53
DFS (smoker)	7	0.61	0.53	0.67
DFS (non-smoker)	6	0.61	0.80	0.46
Alcohol				
OS (alcohol)	7	0.78	0.66	0.87
OS (no alcohol)	(10)	0.46	0.29	0.80
DFS (alcohol)	7	0.71	0.64	0.76
DFS (no alcohol)	(6)	0.56	0.78	0.41
ECS status				
N0				
OS	(8)	0.55	0.49	0.63
DFS	(3)	0.49	1	0.03
N + ECS-				
OS	6	0.78	0.77	0.67
DFS	7	0.78	0.43	1
N + ECS +				
OS	6	0.59	0.74	0.53
DFS	6	0.64	0.81	0.55
Grading				
G1				
OS	(13)	1	1	1
DFS	(13)	0.58	0.33	1
G2				
OS	6	0.61	0.76	0.44
DFS	6	0.58	0.69	0.43
G3				
OS	(9)	0.35	0.09	0.92
DFS	/	0.33	0	1
pT-stage				
pT1/2				
OS	(6)	0.52	0.79	0.29
DFS	(7)	0.52	0.61	0.46
pT3/4a				
OS	6	0.67	0.75	0.54
DFS	6	0.69	0.77	0.56

Optimal cut-off values could not be calculated for all scenarios. In some cases, the AUC was very low, so the cut-off values were not meaningful (e.g. DFS no alcohol, OS N0, OS/DFS pT1/2). When the AUC was < 0.5 the model was insufficient and the cut-off values were deemed to be void (OS no alcohol, DFS N0, OS/DFS G3). In addition, for G1 models, the cut-off values were not reliable because of the low rate of events in this group.

Overall, there were no notable differences between the risk-specific cut-off values, compared with the baseline cut-off values for OS and DFS. There were slightly higher cut-off values for the risk factor alcohol for OS (7 mm, AUC = 0.78) and DFS (7 mm, AUC = 0.71) as well as for N + ECS- for DFS (7 mm, AUC = 0.78) with reasonable AUC.

## Discussion

The use of IFSA is suggested by the current German guidelines for the treatment of OSCC [25]. In this study, defect-driven IFSA provided reliable information about the intraoperative resection margins with a sensitivity of 83.3% and a specificity of 98.6%. Other studies have reported a sensitivity of 45.5%–97% and a specificity of 88.3%–100% [26–28]. Overall, it is possible to make clinical decisions based on the IFSA diagnosis – especially in early stages (T1 and T2) where there is high accuracy. In this investigation, all misclassified resection margins occurred in advanced tumour stages (T3 and T4).

IFSA is not able to provide information regarding the bony resection margins. Nevertheless, our findings imply that if consistent resection in both bony and soft tissue is performed, then IFSA may allow drawing conclusions about the resection status. Of the 52 cases with bone invasion, IFSA was positive for soft tissue margins in 5 (9.6%). This is more than 2.5-fold higher than the rate of positive margins overall. Furthermore, there was a significant difference in the final resection status between patients with or without bone involvement (p = 0.03). These findings imply a tendency for closer margins in patients with bone invasion. A possible reason for this finding might be that gross examination of tumour extension can be performed better in soft tissue than in bone. The increasing usage of computer-assisted planning of resection and reconstruction may be able to improve this point. Indeed, many investigations have shown the benefits of Computer-aided design/Computer-aided manufacturing (CAD/CAM) technology in planning tumour resections as well as the immediate and accurate surgical reconstruction [29–31].

The overall high reliability of IFSA allows the regular performance of reconstructive surgery immediately after tumour resection. Even complex surgical procedures such as microvascular transplants can be performed with a good oncological outcome. In the literature, recurrence rates for immediate versus delayed reconstructive surgery are very similar [32, 33].

However, as IFSA does not directly provide information about the bony resection margins, additional research is needed to establish diagnostic tools that evaluate the intraoperative bony margins. Researchers have described techniques for intraoperative evaluation of bone invasion. Wysluch et al. published the trephine drill technique, with which bone specimens are extracted with a trephine drill from the resected tumour specimen and undergo frozen sectioning. Compared with the definitive histological diagnosis after decalcification process, the trephine drill technique had a consistency of 94% [34]. Another technique is the frozen sectioning of material from bone marrow curettage or inferior alveolar nerve biopsies, as described by Bilodeau et al. for patients with mandibular bone invasion. There were no false-positive findings but the sensitivity was only 50% [35]. Both techniques provide the possibility of an intraoperative evaluation of bony resection margins but leave a need for further investigation to gain a reliable setting.

In general, positive final resection margins are associated with poor OS and DFS [11]. The necessity of initial intraoperative R0 status is still debated. While some studies imply that initial R1 status, even with successfully performed immediate re-resection, decreases OS and DFS [24, 36], there are also studies that have shown that the patient´s prognosis is similar for an R0 status after immediate re-resection compared with an initial R0 status [22, 37]. Ettl et al. found indications that close margins were found more often in tumours which have a tendency for higher differentiation, lymphangiosis and positive neck nodes. Additionally, in their study positive margins revised to negative by immediate re-resection were nevertheless a strong predictor for worse disease-specific survival. Therefore, the authors stated that close margins and especially involved margins are a predictor for local recurrence and thereby, the benefit of frozen sectioning seems questionable to them [38]. In contrast to this, Nentwig et al. found no statistically significant differences between patients with initial R0 resection status versus those with initial R1 status in the IFSA, followed by a successful immediate re-resection. Though, they stated that re-resection failed in 42.1% of the cases with positive margins in the IFSA, with a negative effect on patient´s outcome [22]. The impact of immediate re-resection, induced by positive margins in the IFSA is still debatable. The question of the reliability of matching positive IFSA specimens to the exact intraoperative margins by the surgeon as well as the pathologist is still problematic [39, 40]. Addressing the aspect of improving the communication between surgeon and pathologist, protocols such as the “GAIM” protocol by Tessler et al. may be able to support the interdisciplinary interaction. By using strict inking (different colours for all margins) and analysis strategies for all resection margins (specimen-driven approach, IFSA must include inked an uninked tissue) and systematic revision of the margins if close or involved (entire length of affected margin), they addressed the aspects of reliability and reproducibility. The authors stated that they were able to enhance the precision in margin assessment with their protocol. There is still a need for a long-term follow-up to prove the improvement regarding the outcome, though [41]. Nevertheless, in our study, there were no statistically significant differences between the initial R0 and the R0 after immediate re-resection groups, implying that immediate re-resection based on positive IFSA diagnosis may improve the patient´s prognosis. Due to the low number of intraoperative re-resections, these results should be considered as a tendency. The assessment of close margin status is still debated. German guidelines for the treatment of OSCC suggest adjuvant radiochemotherapy for patients with a close margin status [25]. In some cases with early-stage tumours, depending on the patient´s age, health condition and the tumour characteristics (e.g. perineural or vascular invasion), watchful waiting seems to be a valid treatment alternative [42]. However, Gokavarapu et al. indicated that close margins may have a similar local recurrence rate for early stage OSCCs (pT1N0 /pT2N0). Furthermore, the need for adjuvant therapy in these cases seems to be debatable [43]. In our investigation there was a tendency for a poorer OS and DFS in patients with close margin status, albeit without statistical significance.

Generally, a resection margin of at least 5 mm histologically tumour free tissue is commonly accepted threshold for clear margins [12, 13, 18]. In accordance with this demand, we found cut-off values of 6 mm for OS and DFS. In their recent review, Jang et al. summarised the recent evidence regarding the cut-off values. The authors reported evidence for similar local recurrence rates between the clear and close margin groups in patients with an initial T1/T2 tumour stage. The deep resection margin is especially mentioned to be important for the outcome regarding the local recurrence rate. In addition, tumour thickness has been mentioned as an important risk factor for local recurrence. The authors conclude that there is a need for dynamic resection margins, according to the individual risk factors, including the tumour dimension and the depth of invasion [44]. Also, Kubik et al. found that depending on the margin distance, the presence of PnI seems to increase the risk of locoregional recurrence rates. Especially regarding close resection margins, patients seem to have a higher risk for locoregional recurrence if PnI is present. Their study showed the same risk for locoregional recurrence for patients with PnI- and a resection margin of 2.5 mm and for patients with PnI + and resection margins of 5.0 mm [21]. Furthermore, smoking, alcohol, lymph node involvement with extracapsular spread and histological grade of differentiation are known to be important prognostic factors [45–47]. Therefore, we investigated the impact of these risk factors regarding the resection margin. Finally, our results imply that the presence or absence of these risk factors do not demand different treatment approaches regarding the surgical margin. Based on our data, a histological resection margin of > 5 or > 6 mm seems to be a valid treatment goal, regardless of the accompanying risk factors.

The limitation of this study is the low number of false-positive and false-negative IFSA diagnoses. Therefore, the calculated statistical correlations based on the IFSA should be considered as tendencies and cannot be used to make confirmatory conclusions. Additionally, regarding a median follow-up time of 26 months, it is difficult to make a statement in terms of long-term outcome. In only 11 out of all 213 cases, the follow-up time was at least 50 months. Therefore, the results regarding the long-term outcome should also be considered as a tendency. It would be desirable to confirm these tendencies in further studies. As in all studies dealing with IFSA, it must be mentioned that in the rarest cases, the intraoperative frozen section images the entire resection area of the tumor. Especially the evaluation of the deep margin remains one of the main weaknesses, especially in IFSA with defect-driven approach. Therefore, this limitation should also be considered regarding the assessment of IFSA´s accuracy. The effect of the surgeon's experience on the clinical correlation to the selection of deep-frozen sections is an elusive factor.

## Conclusion

In conclusion, we confirmed that IFSA is a reliable method to assess intraoperative margins. It enables immediate re-resection to achieve an R0 status and/or immediate reconstruction. In cases with bone involvement, additional research is needed to establish a valid method of intraoperative bone assessment. Even in these cases, IFSA can provide helpful information, even though direct bone assessment is not regularly performed. We found that risk factors seemingly do not have a crucial influence on the needed extent of the tumour resection, but additional investigation is needed.

## Data Availability

The data that support the findings of this study are available from the corresponding author upon reasonable request.

## References

[1] van Dijk BA, Brands MT, Geurts SM, Merkx MA, Roodenburg JL (2016) Trends in oral cavity cancer incidence, mortality, survival and treatment in the Netherlands. Int J Cancer 139:574–583. 10.1002/ijc.30107 27038013 10.1002/ijc.30107

[2] Krishna Rao SV, Mejia G, Roberts-Thomson K, Logan R (2013) Epidemiology of oral cancer in Asia in the past decade--an update (2000-2012). Asian Pac J Cancer Prev 14:5567–5577. 10.7314/apjcp.2013.14.10.5567 24289546 10.7314/apjcp.2013.14.10.5567

[3] Listl S, Jansen L, Stenzinger A, Freier K, Emrich K, Holleczek B, Katalinic A, Gondos A, Brenner H, Group GCSW (2013) Survival of patients with oral cavity cancer in Germany. PLoS One 8:e53415. 10.1371/journal.pone.0053415 23349710 10.1371/journal.pone.0053415 PMC3548847

[4] Bernier J, Domenge C, Ozsahin M, Matuszewska K, Lefebvre JL, Greiner RH, Giralt J, Maingon P, Rolland F, Bolla M, Cognetti F, Bourhis J, Kirkpatrick A, van Glabbeke M, European Organization for R and Treatment of Cancer T (2004) Postoperative irradiation with or without concomitant chemotherapy for locally advanced head and neck cancer. N Engl J Med 350:1945–1952. 10.1056/NEJMoa032641 15128894 10.1056/NEJMoa032641

[5] Omura K (2014) Current status of oral cancer treatment strategies: surgical treatments for oral squamous cell carcinoma. Int J Clin Oncol 19:423–430. 10.1007/s10147-014-0689-z 24682763 10.1007/s10147-014-0689-z

[6] O’Brien CJ, Lauer CS, Fredricks S, Clifford AR, McNeil EB, Bagia JS, Koulmandas C (2003) Tumor thickness influences prognosis of T1 and T2 oral cavity cancer--but what thickness? Head Neck 25:937–945. 10.1002/hed.10324 14603454 10.1002/hed.10324

[7] Nishimaki T, Kanda T, Nakagawa S, Kosugi S, Tanabe T, Hatakeyama K (2002) Outcomes and prognostic factors after surgical resection of hypopharyngeal and cervical esophageal carcinomas. Int Surg 87:38–44 12144188

[8] Spiro RH, Guillamondegui O Jr, Paulino AF, Huvos AG (1999) Pattern of invasion and margin assessment in patients with oral tongue cancer. Head Neck 21:408–413. 10.1002/(sici)1097-0347(199908)21:5<408::aid-hed5>3.0.co;2-e 10402520 10.1002/(sici)1097-0347(199908)21:5<408::aid-hed5>3.0.co;2-e

[9] Wong RJ, Keel SB, Glynn RJ, Varvares MA (2000) Histological pattern of mandibular invasion by oral squamous cell carcinoma. Laryngoscope 110:65–72. 10.1097/00005537-200001000-00013 10646718 10.1097/00005537-200001000-00013

[10] Upile T, Fisher C, Jerjes W, El Maaytah M, Searle A, Archer D, Michaels L, Rhys-Evans P, Hopper C, Howard D, Wright A (2007) The uncertainty of the surgical margin in the treatment of head and neck cancer. Oral Oncol 43:321–326. 10.1016/j.oraloncology.2006.08.002 17112772 10.1016/j.oraloncology.2006.08.002

[11] Mannelli G, Comini LV, Piazza C (2019) Surgical margins in oral squamous cell cancer: intraoperative evaluation and prognostic impact. Curr Opin Otolaryngol Head Neck Surg 27:98–103. 10.1097/MOO.0000000000000516 30844923 10.1097/MOO.0000000000000516

[12] Bulbul MG, Zenga J, Tarabichi O, Parikh AS, Sethi RK, Robbins KT, Puram SV, Varvares MA (2021) Margin practices in oral cavity cancer resections: survey of American Head and Neck Society members. Laryngoscope 131:782–787. 10.1002/lary.28976 32827312 10.1002/lary.28976

[13] Meier JD, Oliver DA, Varvares MA (2005) Surgical margin determination in head and neck oncology: current clinical practice. The results of an International American Head and Neck Society Member Survey. Head Neck 27:952–958. 10.1002/hed.20269 16127669 10.1002/hed.20269

[14] Abbas SA, Ikram M, Tariq MU, Raheem A, Saeed J (2017) Accuracy of frozen sections in oral cancer resections, an experience of a tertiary care hospital. J Pak Med Assoc 67:806–809 28507379

[15] Tirelli G, Boscolo Nata F, Gatto A, Bussani R, Spinato G, Zacchigna S, Piovesana M (2019) Intraoperative margin control in transoral approach for oral and oropharyngeal cancer. Laryngoscope 129:1810–1815. 10.1002/lary.27567 30284261 10.1002/lary.27567

[16] Kain JJ, Birkeland AC, Udayakumar N, Morlandt AB, Stevens TM, Carroll WR, Rosenthal EL, Warram JM (2020) Surgical margins in oral cavity squamous cell carcinoma: Current practices and future directions. Laryngoscope 130:128–138. 10.1002/lary.27943 31025711 10.1002/lary.27943

[17] Maharaj DD, Thaduri A, Jat B, Poonia DR, Durgapal P, Rajkumar KS (2021) Performance and survival outcomes of defect-driven versus specimen-driven method of frozen section intraoperative margin assessment in oral cancers. Int J Oral Maxillofac Surg. 10.1016/j.ijom.2021.11.010 10.1016/j.ijom.2021.11.010 34916093

[18] Anderson CR, Sisson K, Moncrieff M (2015) A meta-analysis of margin size and local recurrence in oral squamous cell carcinoma. Oral Oncol 51:464–469. 10.1016/j.oraloncology.2015.01.015 25716108 10.1016/j.oraloncology.2015.01.015

[19] Maxwell JH, Thompson LD, Brandwein-Gensler MS, Weiss BG, Canis M, Purgina B, Prabhu AV, Lai C, Shuai Y, Carroll WR, Morlandt A, Duvvuri U, Kim S, Johnson JT, Ferris RL, Seethala R, Chiosea SI (2015) Early oral tongue squamous cell carcinoma: sampling of margins from tumor bed and worse local control. JAMA Otolaryngol Head Neck Surg 141:1104–1110. 10.1001/jamaoto.2015.1351 26225798 10.1001/jamaoto.2015.1351 PMC5242089

[20] McMahon J, O'Brien CJ, Pathak I, Hamill R, McNeil E, Hammersley N, Gardiner S, Junor E (2003) Influence of condition of surgical margins on local recurrence and disease-specific survival in oral and oropharyngeal cancer. Br J Oral Maxillofac Surg 41:224–231. 10.1016/s0266-4356(03)00119-0 12946663 10.1016/s0266-4356(03)00119-0

[21] Kubik MW, Sridharan S, Varvares MA, Zandberg DP, Skinner HD, Seethala RR, Chiosea SI (2020) Intraoperative margin assessment in head and neck cancer: a case of misuse and abuse? Head Neck Pathol 14:291–302. 10.1007/s12105-019-01121-2 32124417 10.1007/s12105-019-01121-2 PMC7235105

[22] Nentwig K, Unterhuber T, Wolff KD, Ritschl LM, Nieberler M (2021) The impact of intraoperative frozen section analysis on final resection margin status, recurrence, and patient outcome with oral squamous cell carcinoma. Clin Oral Investig 25:6769–6777. 10.1007/s00784-021-03964-y 33956217 10.1007/s00784-021-03964-y PMC8602179

[23] Ribeiro NF, Godden DR, Wilson GE, Butterworth DM, Woodwards RT (2003) Do frozen sections help achieve adequate surgical margins in the resection of oral carcinoma? Int J Oral Maxillofac Surg 32:152–158. 10.1054/ijom.2002.0262 12729775 10.1054/ijom.2002.0262

[24] Szewczyk M, Golusinski W, Pazdrowski J, Masternak M, Sharma N, Golusinski P (2018) Positive fresh frozen section margins as an adverse independent prognostic factor for local recurrence in oral cancer patients. Laryngoscope 128:1093–1098. 10.1002/lary.26890 28988423 10.1002/lary.26890

[25] Wolff KD, Al-Nawas B, Al-Sharif U, Beck J, Bikowski K, Bissinger O, Böhme P, Bönte-Hieronymus I, Bootz F, Bozzato A, Budach W, Burkhardt A, Danker H, Eberhardt W, Engers K, Fietkau R, Frerich B, Gauler T, Gehrmann-Weide K, Germann G, Giannakopoulos N, Gittler-Hebestreit N, Grötz K, Hertrampf K, Hoffmann J, Horch R, Ihrler S, Kaufmann R, Kehrer A, Keilholz U, Klußmann P, Kolk A, Lell M, Lübbe A, Mantey W, Mischkowski R, Moll R, Nieberler M, Nusser-Müller-Busch R, Pistner H, Paradies K, Rau A, Reichert T, Reinert S, Schilling B, Schliephake H, Schmidt K, Schmitter M, Singer S, Terheyden H, Troost E, Waterboer T, Westhofen M, Weitz J, Wirz S, Wittlinger M, Zöphel K (2021) Leitlinienprogramm Onkologie (Deutsche Krebsgesellschaft, Deutsche Krebshilfe, AWMF): S3-Leitlinie Diagnostik und Therapie des Mundhöhlenkarzinoms. Langversion 3.0, 2021, AWMF Registernummer: 007/100OL. https://www.leitlinienprogrammonkologie.de/leitlinien/mundhoehlenkarzinom/ (abgerufen am: 25.06.2023)

[26] Tirelli G, Hinni ML, Fernandez-Fernandez MM, Bussani R, Gatto A, Bonini P, Giudici F, Boscolo Nata F (2019) Frozen sections and complete resection in oral cancer surgery. Oral Dis 25:1309–1317. 10.1111/odi.13101 30933401 10.1111/odi.13101

[27] Buchakjian MR, Tasche KK, Robinson RA, Pagedar NA, Sperry SM (2016) Association of main specimen and tumor bed margin status with local recurrence and survival in oral cancer surgery. JAMA Otolaryngol Head Neck Surg 142:1191–1198. 10.1001/jamaoto.2016.2329 27423460 10.1001/jamaoto.2016.2329

[28] Mair M, Nair D, Nair S, Dutta S, Garg A, Malik A, Mishra A, Shetty Ks R, Chaturvedi P (2017) Intraoperative gross examination vs frozen section for achievement of adequate margin in oral cancer surgery. Oral Surg Oral Med Oral Pathol Oral Radiol 123:544–549. 10.1016/j.oooo.2016.11.018 28159583 10.1016/j.oooo.2016.11.018

[29] Avraham T, Franco P, Brecht LE, Ceradini DJ, Saadeh PB, Hirsch DL, Levine JP (2014) Functional outcomes of virtually planned free fibula flap reconstruction of the mandible. Plast Reconstr Surg 134:628e–634e. 10.1097/PRS.0000000000000513 25357057 10.1097/PRS.0000000000000513

[30] Roser SM, Ramachandra S, Blair H, Grist W, Carlson GW, Christensen AM, Weimer KA, Steed MB (2010) The accuracy of virtual surgical planning in free fibula mandibular reconstruction: comparison of planned and final results. J Oral Maxillofac Surg 68:2824–2832. 10.1016/j.joms.2010.06.177 20828910 10.1016/j.joms.2010.06.177

[31] Wilde F, Hanken H, Probst F, Schramm A, Heiland M, Cornelius CP (2015) Multicenter study on the use of patient-specific CAD/CAM reconstruction plates for mandibular reconstruction. Int J Comput Assist Radiol Surg 10:2035–2051. 10.1007/s11548-015-1193-2 25843949 10.1007/s11548-015-1193-2

[32] Hanken H, Wilkens R, Riecke B, Al-Dam A, Tribius S, Kluwe L, Smeets R, Heiland M, Eichhorn W, Grobe A (2015) Is immediate bony microsurgical reconstruction after head and neck tumor ablation associated with a higher rate of local recurrence? J Craniomaxillofac Surg 43:373–375. 10.1016/j.jcms.2015.01.006 25701393 10.1016/j.jcms.2015.01.006

[33] Mucke T, Wolff KD, Wagenpfeil S, Holzle F, Scholz M (2010) Reliability of near-infrared angiography and micro-Doppler sonography for evaluating microvascular anastomoses. Plast Reconstr Surg 126:1506–1514. 10.1097/PRS.0b013e3181f0215a 21042107 10.1097/PRS.0b013e3181f0215a

[34] Wysluch A, Stricker I, Holzle F, Wolff KD, Maurer P (2010) Intraoperative evaluation of bony margins with frozen-section analysis and trephine drill extraction technique: a preliminary study. Head Neck 32:1473–1478. 10.1002/hed.21350 20187018 10.1002/hed.21350

[35] Bilodeau EA, Chiosea S (2011) Oral squamous cell carcinoma with mandibular bone invasion: intraoperative evaluation of bone margins by routine frozen section. Head Neck Pathol 5:216–220. 10.1007/s12105-011-0264-0 21512783 10.1007/s12105-011-0264-0 PMC3173530

[36] Patel RS, Goldstein DP, Guillemaud J, Bruch GA, Brown D, Gilbert RW, Gullane PJ, Higgins KM, Irish J, Enepekides DJ (2010) Impact of positive frozen section microscopic tumor cut-through revised to negative on oral carcinoma control and survival rates. Head Neck 32:1444–1451. 10.1002/hed.21334 20091833 10.1002/hed.21334

[37] Kwok P, Gleich O, Hubner G, Strutz J (2010) Prognostic importance of “clear versus revised margins” in oral and pharyngeal cancer. Head Neck 32:1479–1484. 10.1002/hed.21349 20175196 10.1002/hed.21349

[38] Ettl T, El-Gindi A, Hautmann M, Gosau M, Weber F, Rohrmeier C, Gerken M, Muller S, Reichert T, Klingelhoffer C (2016) Positive frozen section margins predict local recurrence in R0-resected squamous cell carcinoma of the head and neck. Oral Oncol 55:17–23. 10.1016/j.oraloncology.2016.02.012 27016013 10.1016/j.oraloncology.2016.02.012

[39] Kerawala CJ, Ong TK (2001) Relocating the site of frozen sections--is there room for improvement? Head Neck 23:230–232. 10.1002/1097-0347(200103)23:3<230::aid-hed1023>3.0.co;2-v 11428454 10.1002/1097-0347(200103)23:3<230::aid-hed1023>3.0.co;2-v

[40] Olson SM, Hussaini M, Lewis JS Jr (2011) Frozen section analysis of margins for head and neck tumor resections: reduction of sampling errors with a third histologic level. Mod Pathol 24:665–670. 10.1038/modpathol.2010.233 21217647 10.1038/modpathol.2010.233

[41] Tessler I, Marilena V, Alon EE, Gecel NA, Remer E, Gluck I, Yoffe T, Dobriyan A (2023) Paradigm change for intraoperative surgical margin assessment for oral squamous cell carcinoma. Laryngoscope. 10.1002/lary.31126 10.1002/lary.31126 37929854

[42] Dik EA, Willems SM, Ipenburg NA, Adriaansens SO, Rosenberg AJ, van Es RJ (2014) Resection of early oral squamous cell carcinoma with positive or close margins: relevance of adjuvant treatment in relation to local recurrence: margins of 3 mm as safe as 5 mm. Oral Oncol 50:611–615. 10.1016/j.oraloncology.2014.02.014 24630900 10.1016/j.oraloncology.2014.02.014

[43] Gokavarapu S, Chander R, Parvataneni N, Puthamakula S (2014) Close margins in oral cancers: implication of close margin status in recurrence and survival of pT1N0 and pT2N0 oral cancers. Int J Surg Oncol 2014:545372. 10.1155/2014/545372 25436146 10.1155/2014/545372 PMC4244693

[44] Jang JY, Choi N, Jeong HS (2022) Surgical extent for oral cancer: emphasis on a cut-off value for the resection margin status: a narrative literature review. Cancers (Basel) 14. 10.3390/cancers14225702 10.3390/cancers14225702 PMC9688090 36428794

[45] Kademani D, Bell RB, Bagheri S, Holmgren E, Dierks E, Potter B, Homer L (2005) Prognostic factors in intraoral squamous cell carcinoma: the influence of histologic grade. J Oral Maxillofac Surg 63:1599–1605. 10.1016/j.joms.2005.07.011 16243176 10.1016/j.joms.2005.07.011

[46] Almangush A, Makitie AA, Triantafyllou A, de Bree R, Strojan P, Rinaldo A, Hernandez-Prera JC, Suarez C, Kowalski LP, Ferlito A, Leivo I (2020) Staging and grading of oral squamous cell carcinoma: an update. Oral Oncol 107:104799. 10.1016/j.oraloncology.2020.104799 32446214 10.1016/j.oraloncology.2020.104799

[47] Shaw RJ, Lowe D, Woolgar JA, Brown JS, Vaughan ED, Evans C, Lewis-Jones H, Hanlon R, Hall GL, Rogers SN (2010) Extracapsular spread in oral squamous cell carcinoma. Head Neck 32:714–722. 10.1002/hed.21244 19827119 10.1002/hed.21244

